# Maintaining vocational education: strategies of a public institution aimed at adolescents in social vulnerability

**DOI:** 10.1590/0034-7167-2023-0285

**Published:** 2025-06-20

**Authors:** Lilian Dias Ennes, Camila Pureza Guimarães da Silva, Tânia Cristina Franco Santos, Patrícia dos Santos Augusto, Gabriella Picoli dos Santos Faustino, Antonio José de Almeida

**Affiliations:** I Hospital Federal Cardoso Fontes. Rio de Janeiro, Rio de Janeiro. Brazil; II Universidade Federal do Rio de Janeiro. Rio de Janeiro, Rio de Janeiro. Brazil

**Keywords:** Adolescent, Professional Training, Professional Education, History of Nursing, Nursing., Adolescente, Capacitación Profesional, Educación Profesional, Historia de La Enfermería, Enfermería.

## Abstract

**Objectives:**

to analyze the political and administrative strategies for maintaining the courses at the Healthcare Professional Unit and the *Hospital da Criança e do Adolescente* Study Center of the Brazilian Center for Childhood and Adolescence Foundation.

**Methods:**

a historical study, with sources consisting of journalistic articles and official documents, in addition to eight participants. Data were collected from May to December 2021 and analyzed based on articles on vocational education and educational policies at national and state levels. The time frame covers the years 1990 to 1997.

**Results:**

strategies adopted by workers culminated in changes at the *Centro Piloto Quintino*, with the structuring of the Comprehensive Learning Center, and the maintenance of courses at the Healthcare Professional Unit.

**Final Considerations:**

the strategies resulted in the restructuring and redefinition of the space that was previously intended to stigmatize adolescents in situations of social vulnerability.

## RESUMEN

**Objetivos:**

analizar las estrategias políticas y administrativas para el mantenimiento de los cursos de la Unidad Profesional del Área de Salud y del Centro de Estudios del Hospital del Niño y del Adolescente de la Fundación Centro Brasileño de la Infancia y del Adolescente.

**Métodos:**

estudio histórico, con fuentes compuestas por artículos periodísticos y documentos oficiales, además de ocho participantes. Los datos se recopilaron de mayo a diciembre de 2021 y se analizaron en base a artículos sobre educación vocacional y políticas educativas a nivel nacional y estatal. El período abarca los años 1990 a 1997.

**Resultados:**

las estrategias adoptadas por los trabajadores culminaron en cambios en el *Centro Piloto de Quintino*, con la estructuración del Centro de Educación Integral, y en el mantenimiento de cursos en la Unidad Profesional del Área de Salud.

**Consideraciones Finales:**

las estrategias resultaron en la reestructuración y Resignificación del espacio que antes pretendía estigmatizar a los adolescentes en situación de vulnerabilidad social.

## INTRODUCTION

The 1989 presidential election brought a paradox to studies of Brazilian politics, as it was remembered for the country’s long journey of democratic transition and the uncertainty in the new electoral scenario experienced at the time. Fernando Afonso Collor de Mello took over the Presidency of Brazil on March 15, 1990 (until 1992), this being the first presidential election by direct vote, after 21 years under the military dictatorship and four years after the promulgation of the Constitution of the Federative Republic of Brazil, which took place on October 5, 1988^(1,2)^. Collor took office as president in the midst of a major economic and social crisis that the country was going through.

Fernando Collor government’s plan to reduce inflation rates included goals such as state, monetary, fiscal and administrative adjustments, with the proposal of reducing bureaucracy and the public sector size. The extinction and dissolution of federal public administration entities, which were reaffirmed through Law 8,029 of April 12, 1990, are among the president’s first acts^(3,4)^.

Thus, the Executive Branch was authorized to extinguish or transform some public entities that were in force until then. Among them was the Brazilian National Foundation for the Welfare of Minors (In Portuguese, *Fundação Nacional do Bem-Estar do Menor*- FUNABEM), established by Law 4,513 of December 1, 1964, which was renamed the Brazilian Center for Childhood and Adolescence Foundation (In Portuguese, *Fundação Centro Brasileiro para Infância e Adolescência*- FCBIA) by Law 8,029/1990^(5-7)^.

FUNABEM was created in the same year that the military regime was established in Brazil, and its policy of internment to resolve the problem of minors was considered a milestone for the institution, since it was based on the doctrine of national security, employed by the Higher School of War (In Portuguese, *Escola Superior de Guerra* - ESG). At the time, intense debates were sparked about the creation of a management entity for children and youth that would eradicate the former Child Welfare Service (In Portuguese, *Serviço de Assistência ao Menor* - SAM), an institution that preceded FUNABEM^(8)^.

FCBIA, subordinate to the Ministry of Social Action (MSA), aimed to formulate, standardize and coordinate the policy for the defense of the rights of children and adolescents. This change in name was a strategy to redefine the attention given to this public, as there was significant pressure from society and international organizations, such as the influence of guidelines on children and adolescents, promulgated by the United Nations (UN), which drove this change^(7)^.

Thus, FCBIA was part of a framework of laws that preceded the Statute of Children and Adolescents (In Portuguese, *Estatuto da Criança e do Adolescente* - ECA), defined by Law 8,069 of July 13, 1990^(9)^, which provides for comprehensive protection of children and adolescents. Before ECA, the term “minor” was used to refer to individuals whose basic needs were not met, and the economic factor was considered one of the main reasons that led boys and girls to be confined in care institutions, where they remained for an indefinite period of time^(10)^.

The administrative headquarters of FCBIA was located in Rio de Janeiro, as it was the Federal District for a long period, but the foundation had several units throughout the national territory, both state-owned and privately affiliated units^(7)^. However, after the establishment of ECA, this socio-educational model of institutionalizing children and adolescents weakened.

The authorities’ perception of the costs of maintaining this precarious structure and the constant criticism from society, which called for decent care for young people under the yoke of the State, contributed to the downfall of FCBIA in 1995. Thus, Fernando Henrique Cardoso, upon taking over the Presidency of the Republic (1995-1999), extinguished FCBIA by Provisional Measure 813, of January 1, 1995^(9,10)^.

The closure of FCBIA did not occur immediately after the processing of Provisional Measure 813, as published by the newspaper *Folha de São Paulo* on January 2, 1995, in a report entitled: “*Legião Brasileira da Boa Vontade e FCBIA deixam de existir de forma gradual*”. It was necessary to ensure that programs and agreements were not interrupted in the process of transferring functions to other ministries^(10,11)^.

Thus, professionals who were not in the process of retiring or did not request voluntary resignation at the time would be gradually transferred to other federal agencies. The implications of the end of FCBIA’s activities had repercussions on federal employees’ lives as well as on the reorganization of spaces and the allocation of assets left by the foundation throughout Brazil^(10)^.

It is worth noting that this context had an impact on the *Hospital da Criança e do Adolescente* (HCA) Study Center of FCBIA, a department authorized to provide professional training courses in health, according to Opinion52/91 of the State Education Council of Rio de Janeiro (In Portuguese, *Conselho Estadual de Educação do Rio de Janeiro* - CEE/RJ). The courses offered included nursing technician, nursing assistant, clinical pathology assistant, clinical pathology technician, dental office attendant, prosthesis laboratory assistant, dental prosthesis laboratory technician and pharmacy assistant^(11)^.

Regarding professional nursing courses, despite the authorization of the nursing technician course, only the nursing assistant course was offered, and remained so even after the closure of FCBIA activities. The nursing assistant course was maintained after strategies adopted by professors, students and other education workers, who were dissatisfied with the impact that the interruption of the course would have on workers and adolescents in the field, who saw in that educational unit an opportunity for professional qualification. This situation resulted in the maintenance of courses at the Healthcare Professional Unit (In Portuguese, *Unidade Profissionalizante da Área da Saúde* - UPAS), as a result of the new educational policy implemented in the State of Rio de Janeiro, Brazil, with the creation of a Comprehensive Learning Center (CLC) in the educational complex called “*Centro Piloto de Quintino*”.

Considering the above, we present the following investigative question: how did the strategies adopted by professors and political and administrative authorities impact the maintenance of courses at UPAS and the HCA Study Center at FCBIA after the extinction of this foundation?

This study is justified by the academic and social relevance involved in vocational education aimed at adolescents in social vulnerability, especially when such an initiative is driven, at the same time, by vocational educational policies at federal and state levels, in this specific case, the state of Rio de Janeiro.

## OBJECTIVES

To analyze the political and administrative strategies for maintaining the courses at UPAS and the HCA Study Center at FCBIA.

## METHODS

### Ethical aspects

Ethical aspects were respected, in accordance with recommendations of Resolutions 466/12 and 510/16 of the Brazilian National Health Council. For the interviews, all participants signed an Informed Consent Form. The study was approved by the signatory institution’s Research Ethics Committee.

### Study design

This is a historical study, from the perspective of the history of the present time, whose *corpus* was composed of direct (written and oral) and indirect historical sources. The time frame covers the years 1990 to 1997, a period in which laws relevant to the context of this study were published. The initial historical milestone is defined as Law 8,029 of April 12, 1990, which changed the name of FUNABEM to FCBIA. This law also greatly modified the structure and operation of the hospital and the FCBIA Study Center and, thus, the professional qualification courses in health, such as the nursing assistant course. The final milestone was defined in 1997, when the HCA physical structure, part of the extinct FCBIA, incorporated UPAS. The COnsolidated criteria for REporting Qualitative research (COREQ) guidelines were followed.

### Study setting

The research setting was the *Centro Piloto de Quintino*, whose territorial extension is 1,900,000 m^2^. For decades, this space belonged to the Federal Government, being ceded, through an agreement, to the Government of the State of Rio de Janeiro, which transformed it into an important center for the development of public policy for professional education, under the management of Governor Marcello Nunes de Alencar (1995-1999), through the Public School Support Foundation (In Portuguese, *Fundação de Apoio à Escola Pública* - FAEP), subordinated to the State Department of Education (In Portuguese, *Secretaria Estadual de Educação* - SEEDUC), from July 1987 to February 1996^(12)^.

### Data collection and organization

Data collection for the documentary corpus took place from May to December 2021. The direct written historical sources consisted of laws, decrees, ordinances, regulatory projects for the nursing assistant course, *Revista Brasil Jovem*, and an article in *Folha de São Paulo*. The documentary *corpus* was compiled through searches on government websites, in institutional archives, the setting for this research, and in the personal archives of the participants in this study. The indirect sources consisted of articles on the subject.

Documents that addressed the daily lives of health and education workers who experienced the maintenance of the nursing assistant course at the HCA Study Center, in addition to those related to educational policy in the state of Rio de Janeiro, were included. Textual sources damaged by various reasons, such as humidity, mold, termites, moths, among others, which made the document illegible, were excluded.

Direct oral sources consisted of interviews with eight participants, including: pedagogue (1); nursing professor (2); instructor (1); student (1); biology professor (1); student inspector (2). All were over 50 years old, five were female and three were male. In relation to education, six had completed undergraduate studies and two had completed high school. Regarding employment status, one was a federal civil servant, five were state civil servants, one had a temporary contract and one had no employment relationship with the institution.

The technique used was thematic oral history^(13)^. To select participants, we used the nominal list of professionals who worked or studied in professional health courses at the HCA Study Center during the study period. Participants who worked or studied in professional health courses at the HCA Study Center during the study period were included. Memory impairment or other physical or cognitive impairment that prevented the interview from being conducted were excluded.

The interviews were collected after scheduling via email, telephone calls and searching for professionals in their respective fields of professional activity. Only after participants agreed to participate in the research were the interviews scheduled, according to the availability of each participant, lasting between 40 minutes and 1 hour and 30 minutes. All potential participants contacted and who agreed to participate in the research were interviewed.

It is worth noting that, due to the novel coronavirus pandemic (SARS-CoV-2 (COVID-19)), and aiming at ensuring social distancing for those participants who have difficulty moving around or are considered at risk, the interviews were conducted and recorded virtually through a Google Meet access link sent to participants minutes before the interview. Only one participant requested that the interview be held in person. In both types of interviews, any questions about the Informed Consent Form (ICF), sent in advance, also virtually, were read and clarified, as well as about the acceptance to participate in the recording proposed by the study.

In order to guarantee anonymity, participants were identified by the letter P, followed by a cardinal number, according to the order of interviews.

### Data analysis

The data were organized, classified and analyzed in accordance with the historical method. For the documentary *corpus* analysis, the written sources were catalogued in chronological sequence, and, subsequently, external and internal criticism was carried out, in order to guarantee the documents’ authenticity, legitimacy, veracity and reliability. In this way, the systematic exposure of historiographical texts to reader and the critical and analytical use of these were consolidated as one of the main constitutive factors in the affirmation of the authenticity of the historical fact^(14)^.

As for the direct oral sources, the interviews were transcribed and validated by participants. From then on, the documentary *corpus* was subjected to active procedures of interrogation of documents, with an independent stance from the official version, allowing for better evidence of the historical phenomenon. The reliability of results was ensured by valuing the documentary set, and not the documents in isolation, establishing the following axes: Changes in the *Centro Piloto de Quintino* with the structuring of the Comprehensive Learning Center; Maintaining the courses at the Healthcare Professional Unit at the Comprehensive Learning Center.

The data were analyzed based on articles on vocational education and educational policies at national and state levels in the LILACS, MEDLINE, SciELO and *BVS Enfermagem* indexing databases, useful for understanding the context in which the sources were produced, aiming to understand the discourse contained in the texts, i.e., the indirect sources.

## RESULTS

### Changes in the *Centro Piloto de Quintino* with the structuring of the Comprehensive Learning Center

The political and social changes that occurred during the first term of President Fernando Henrique Cardoso (1995-1999) had a significant impact on the world of work, health and education, in line with the neoliberal thinking prevailing at the time, which advocated state intervention in the economy, with extensive privatization of public services. Despite criticism from some educators, an intense reorganization of the educational systems was sought, which should respond to the logic of market demands^(4)^.

Among these changes was the gradual closure of FCBIA’s activities in the 1990s. To this end, however, Agreement 034-A/00/93, dated August 10, 1993, was signed between the Federal Government and the Government of the State of Rio de Janeiro, with the aim of assuming a commitment to mutual cooperation for the development of integrated actions and the implementation of socio-educational protection measures aimed at children and adolescents in situations of personal and social risk, with a focus on the units that make up the educational complex called “*Centro Piloto de Quintino*”^(15)^.

In this context of changes, educator Nilda Teves Ferreira (1941-2022) stood out, project author and CLC coordinator, created by Decree21,752 of November 8, 1995, linked to the Civilian Office. CLC would function as the institution that would assume the socio-educational actions of the extinct FCBIA, through a proposal to offer professional courses integrated with sports practice, in a pedagogical logic very similar to that of the Integrated Centers of Public Education (In Portuguese, *Centros Integrados de Educação Pública* - CIEP), created in the first term of Governor Leonel Itagiba de Moura Brizola (1991-1994)^(12,16)^.

As for CLC, Nilda Teves Ferreira confirmed, in an interview conducted in 2012, and published in the International Journal of Phenomenology, Hermeneutics and Metaphysics in 2022^(17)^, that:

[...] *after winning over the employees who were accustomed to the old regime, we began to adapt the buildings and facilities to suit our purpose. We intended to build what was called CLC, the Comprehensive Learning Center. We had a big challenge ahead of us, because the whole place had a very negative image... it was dark, had high walls and gates like a custodial home, and had trees with fallen branches* [...].

According to the educator, in an article she wrote, entitled “*Projeto educacional para mega-cidade: relato de caso*”, her experience in education would have brought the necessary strength and self-esteem to the changes in that space, called by Nilda Teves Ferreira herself as an “educational shopping mall”, considering that there were countless opportunities for a new generation of children and adolescents, putting an end to the stigma caused by the old correctional center for minors^(17,18)^.

This recognition could be observed according to fragments of interviews with CLC employees:

[...] *at that time, we had a pioneer at UFRJ, Nilda Teves. Nilda Teves was a person who believed. She had a PhD from the UFRJ School of Education and believed in this very much. Nilda* [Teves] *was one of the presidents, the first president of FAEP, who created the entire framework for FAEP* [...]. (P1)

[...] *I have no words to express the valuable contribution of Professor Nilda* [Teves] *to the reactivation, the Centro Piloto de Quintin orevitalization. So, my words to Professor Nilda are of gratitude* [...]. (P2)

[...] *that woman* [Nilda Teves] *is fantastic* [...] *I’m a fan of hers* [...] *fantastic, fantastic, fantastic. Marcello de Alencar* [governor], *every month, he would go to Quintino four or five times just to visit her and see how things were there. They were very good friends, very close.* (P3)

[...] *CLC project by Professor Nilda* [Teves] *was very well received, very well received by the community, because they saw the possibility of resuming activities that they had access to. Like the sports part, for instance, and some pedagogical activities.* (P5)

The political environment was then in place for Nilda Teves to be invited by Governor Marcello de Alencar to occupy, between 1995 and 1996, the position of coordinator of CLC and, at the same time, president FAEP^(12)^.

However, Nilda Teves Ferreira had to face the opposition of some federal employees and other workers with different employment relationships, who felt threatened. What caused these employees’ concern was the fear of losing their employment relationships, since they were outsourced. There was also the fact that they did not accept the departure of children and adolescents who remained in the FCBIA-CLC transition center to other care institutions. This situation led them to organize themselves to confront what they considered to be neglect by the authorities. Thus, they participated, together with students, in a quick protest, as described by a contracted employee:

[...] *the protest didn’t even last half an hour, you understand? Students sat on the ground, everyone sat on the ground on Rua Clarimundo de Melo, and we paralyzed it for a while, and the police arrived* [...]*. The police took us off the street, put the students inside and even threatened us, because there were minors and we were going to answer for the minors who were there* [...]. (P4)

The precarious employment relationship of outsourced workers - those who were not public employees and therefore did not enjoy job stability - did not allow them to move forward in a movement to fight for the maintenance of their jobs. On this subject, Nilda Teves Ferreira confirmed^(17)^ that:

[...] *with the development of schools and vocational training, the institution’s technicians came to the conclusion that it was not possible to serve around four thousand students and maintain the residents. This was a very radical decision. The Foundation for Childhood and Adolescence* [FIA] *would be responsible for housing, and CLC would be responsible for education, including for the residents.* [...] *CLC became a successful project, to the point that it opened space to join technical schools* [...].

In this context, the educator understood that she was clarifying doubts about the educational project she proposed, which envisioned the possibilities of creating a new instruction of welcome through education, considering the process of redemocratization and the need to confront an entire retrograde political culture. Thus, Nilda Teves Ferreira stated that:

*You donot go from the old to the new all at once! You donot slide from one culture to another overnight* ^(17)^.

### Maintaining the courses at the Healthcare Professional Unit at the Comprehensive Learning Center

In order to grant greater administrative autonomy to CLC, Governor Marcello de Alencar enacted Decree 22,011, on February 9, 1996, separating the Center of the Civil Office and subordinating it to FAEP. Another seven state technical schools that had previously been linked to SEEDUC were also transferred to FAEP. At the time, FAEP ceased to be subordinate to SEEDUC and became linked to the State Department of Science and Technology (In Portuguese, *Secretaria Estadual de Ciência e Tecnologia* - SECT), which represented an important initiative for professional education in the state^(16,19,20)^. By this decree, some professionals in the education field who were of interest to CLC management were appointed and remained assigned to the *Centro Piloto de Quintino*.

One of the challenges faced by educator Nilda Teves Ferreira in managing CLC was the fact that she found that the *Centro Piloto de Quintino* offered healthcare courses, including nursing assistant courses, that were left over from HCA. Some of these courses had been interrupted due to the closure of FCBIA and the consequent transfer of its professionals to other federal agencies. The nursing assistant course would be resumed in an administrative area of CLC, where the FAEP/CLC management team would be located, as described by a study participant:

[...] *when we took over* [tenured professors]*, we were then directed to the Study Center* [...] *and informed that we would take over classes in the nursing assistant course that had been paralyzed since the* [HCA] *hospital had been deactivated.* [...] *the theoretical classes would take place in a space in the building in the foundation’s presidency* [...]. (P5)

It should be noted that, according to the Brazilian Federal Court of Auditors (In Portuguese,*Tribunal de Contas da União* - TCU 012.096/1996-2)^(21)^, the deactivation of HCA had already been verified on March 29, 1995. Prior to this event, professional training courses in health were taught on its premises, more specifically by the HCA Study Center. Thus, when the first tenured professors arrived, they would take over the classes that were in this administrative building of CLC, since the professors who preceded them, nurses assigned to HCA, had already been transferred or retired.

The set of these courses became known as UPAS. UPAS became responsible for offering courses at the elementary and secondary levels in health at the *Centro Piloto de Quintino*. Thus, pedagogue Celso do Nascimento Faustino (1951-2022), a federal public servant, was appointed, according to Decree 22,011, 1996(19), to take over UPAS management. Regarding this, he himself stated:

[...] *there was a change in the administrative structure of FCBIA* [...] *and the health courses were transformed and became part of the Healthcare Professional Unit, and this name was given by the administrative team and the professors*[...]. (P2)

Pedagogue Celso do Nascimento Faustino, having demonstrated important cultural and scientific capital, in addition to his interest in maintaining professional training courses in health, had been invited by CLC management team, through Nilda Teves de Ferreira, to take over the UPAS management. Concerning his professional career as a federal public servant at the HCA Study Center, Celso do Nascimento Faustino reported:

[...] *due to the need to reactivate the operation of professional qualification courses at elementary and secondary levels in the health area at the* [HCA]*Study Center, I was administrative secretary, later becoming school secretary, course coordinator and director of UPAS in 1996* [...]. (P2)

Committed to UPAS, pedagogue Celso Faustino accepted the position of director, being one of the last remaining employees of HCA, familiar with the structure and operation of the courses offered. Moreover, CLC implementation brought greater visibility to the courses offered throughout the *Centro Piloto de Quintino*, which were publicized more widely during Nilda Teves’s administration. It is worth noting that, although UPAS had been recognized by its peers since 1996, it was only in 2001 that it was legitimized as a teaching unit by Ordinance PR/FAETEC 79 of February 19^(21-23)^.

Thus, excited about the increase in demand for courses, and considering that the nursing assistant course should be of greatest interest to society at that educational institution, Celso Faustino considered with Nilda Teves the possibility of occupying the hospital, which had been deactivated until then, to house UPAS permanently. Thus, the director tried to persuade CLC coordinator, as per the following excerpt from the interview:

[...] *I’m always pressuring Professor Nilda and appealing to occupy the hospital* [HCA]*, but I’m not going to occupy everything, I’m just going to occupy the first floor and it will be possible for us to organize the courses* [...]. (P2)

Although it was a demand from professors and students, it is worth noting that the director of UPAS, despite enjoying prestige with the president of FAEP and coordinator of CLC, responsible for managing that large area of the *Centro Piloto de Quintino*, understood that conquering the space of HCA to transform it into a teaching unit would not be a simple task. This was because a considerable amount of federal assets still remained in the hospital, which required measures to address bureaucratic obstacles, with a view to relocating them. Despite Decree of August 27, 1996, the completion of the inventory work related to the assets left by the extinct FCBIA was recorded^(10,24)^.

In November 1996, Decree 2,059 established the transfer of the movable assets of the former FCBIA to the Ministry of Justice, and it was up to this ministry to indicate which agencies and entities would receive the assets. Thus, as provided for in Article 2 of Decree 2,059, Minister of Justice Nelson Azevedo Jobim (1995-1997) authorized the allocation of movable assets that were not used in his services, i.e., they could be sold, transferred or donated to the states, the Federal District and the municipalities^(10,25)^.

The director of UPAS also discussed with Nilda Teves the need to offer health courses to adolescents from the Quintino Bocaiúva region who had less education, as he was familiar with the reality of that area. Finally, in 1997, Nilda Teves agreed with the director of UPAS to occupy the first floor of the HCA building and begin the transition of health courses to that space.

## DISCUSSION

In Brazil, the ideals of a neoliberal policy, initiated by Fernando Collor de Mello and consolidated by Fernando Henrique Cardoso, set the tone for a policy that sought to reduce public spending by reducing the payroll of federal public servants and privatizing state-owned companies, which were essential focuses of his government plans. To this end, important legal provisions were created to support his actions, such as Law 8,029 of April 12, 1990, which led to the change in the name of FUNABEM to FCBIA, proposing a change in the policy of care for children and adolescents.

However, the study showed, in practice, that no significant changes were observed in the care provided to this public. This change in name initially calmed an outcry from society due to the many criticisms that had been leveled at the Brazilian Federal Government for Brazil’s participation in the Convention on the Rights of the Child adopted by the UN General Assembly on November 20, 1989^(26)^.

It is possible to understand the ineffectiveness of this initiative, since a distorted perception still prevailed in both society and the Brazilian State, in which individuals who lacked solidarity and assistance were treated as individuals who should be the target of surveillance and repression. Hence, public safety issues related to minors were commonly associated with a portion of children and adolescents who had not achieved success due to “dropping out of school, informal and/or early work, or improper socialization (such as involvement with drug use and trafficking)”. This social group reflected an individual who required attention from the State, since the family had not satisfactorily fulfilled its role of guiding them to adulthood and living in society, which therefore justified State intervention^(8)^.

This social structure reveals and, at the same time, consolidates a conservative way of thinking in Brazilian society that contributes to the construction and maintenance of social stigmas directed at people involved in criminal acts. This partly explains why this group is stigmatized by society in general, which reacts and reduces the discussion of the problem to retrograde issues such as lowering the age of criminal responsibility. As a result, these young people are the target of prejudice and social isolation^(27)^.

The political and social changes that occurred in the world in the 1990s had a major influence on actions to protect the rights of children and adolescents, which were initiated by international organizations that dictated rules based on the importance of deinstitutionalization and more dignified care for the less privileged of the child and adolescent population. In Brazil, during a period marked by the resumption of the democratic constitutional order, Law 8,069 of 1990, known as ECA, was enacted. ECA outlined new paradigms for managing a social group so affected by structural inequalities that exist in the country^(27)^.

The study showed the importance of ECA by highlighting two important articles of the 1988 Constitution for the recognition of children and adolescents as subjects of rights: Art. 227, when describing the rights to life, health, food, education, leisure, professionalization, culture, dignity, respect, freedom and family and community life; and Art. 228, which makes important reference to criminal non-imputability of minors under 18 years of age, in addition to both being understood as determinants of the legal device that meets children’s and adolescents’ needs^(10)^.

Five years later, Fernando Henrique Cardoso dissolved FCBIA through Provisional Measure 813 of January 1, 1995. This measure caused dissatisfaction among many federal employees in Rio de Janeiro, where it was headquartered. As a result of this closure, they were gradually transferred. The emptying of the site sparked speculation, since it was a large area of approximately 1,900,000 m^2^, known as the *Centro Piloto de Quintino*. Additionally, other factors that led to the closure of FCBIA and its affiliated institutions throughout the country were high maintenance costs, suspicions of fraud and corruption, and the social disapproval that the institution had been causing^(10)^.

Regardless of how the *Centro Piloto de Quintino* was configured, it is worth highlighting that it was transformed into an important center for professional education by the articulate and competent educator Nilda Teves Ferreira, who faced discredit from some, but managed, through the political support of Governor Marcello Alencar, to develop the project called CLC, created by Decree 21,752 of November 8, 1995, initially linked to the Civilian Office.

Nilda Teves Ferreira intended to transform that scenario, which maintained, in the collective imagination of professionals, students and the surrounding population, stigmatizing memories of hardships experienced by children and adolescents who had once been in that space. To this end, it is important to consider the recognition of the different agents who worked with Nilda Teves, seeing her as a reference in the field of education in the state of Rio de Janeiro.

Pedagogue Celso do Nascimento Faustino, a federal civil servant who was familiar with the history of the FCBIA Study Center, having served as administrative secretary during the period in which HCA and the Study Center - structures linked to FCBIA - were still active, was invited and appointed as director of UPAS by the director of CLC at the time. This civil servant also stood out for having led the defense of the continuity of professional training courses in health, including the nursing assistant course, considered by him to be the most relevant of UPAS courses.

It is worth noting that, during the period when the name was changed from FUNABEM to FCBIA until the closure of its activities in 1995, the courses that were held at the HCA facilities were no longer held. In this scenario, pedagogue Celso do Nascimento Faustino, in addition to professors and students from UPAS, made efforts to continue the courses and their implementation at the HCA facilities, which were already deactivated. All the assets and documentation pertinent to the period in which it was active remained in this space. This storage made it difficult to occupy them.

The understanding of the importance of occupying the old building - which had long housed HCA - led to a struggle by employees to have health courses offered in this space. The location was considered the most appropriate for basic and secondary level professional training in health, and its acquisition represented a symbolic asset for professionals in the different courses.

Even with Nilda Teves’s prestige, pedagogue Celso Faustino knew that freeing up the space of HCA would not be easy, due to all the assets that had been left inside. Although a decree of August 27, 1996 recorded the completion of the inventory work related to these assets, the new Decree 2,059 of November 5, 1996, designated the transfer of the movable assets of the extinct FCBIA to the Ministry of Justice, and it was up to the Ministry of Justice to indicate which agencies and entities would be granted the furniture^(10,25)^.Finally, in 1997, Nilda Teves authorized the director of UPAS to occupy the first floor of the HCA building and begin the transition of health courses to that space.

This led to the resumption of educational activities aimed at the professionalization of adolescents and young people at the *Centro Piloto de Quintino* and, consequently, the recovery of the perspective of professionalization of that group. To better understand the relevance of this initiative, it is worth recalling the signing of Law 8,080 of September 19, 1990, which implemented the Brazilian Health System (In Portuguese, *Sistema Único de Saúde* - SUS), which ensured comprehensive healthcare as a right for all Brazilians. Regarding the nursing assistant course, one of the most sought after by young students at the *Centro Piloto de Quintino*, the search for professional qualification in the job market was also justified by the contribution to SUS maintenance and development, as it is the largest contingent among healthcare professionals working in Brazil^(28,29)^.

The importance of professional qualifications for adolescents and young people is successful in other countries that have adopted professionalization initiatives aimed at this population group as a social development policy. Studies involving 40 countries and coordinated by the Organization for Economic Cooperation and Development (OECD) in 2013 show that Brazil was among the countries with low participation of young people in professional education courses. Data from 2012 reveal that, among students enrolled in the secondary education system (general or technical), 14% were enrolled in Vocational and Technological Education (VTE) and, in 2015, only 9%. However, in other OECD member countries, this percentage reaches 70% of young people enrolled in the VTE program^(30)^.

According to this OECD study, it is possible to highlight a significant contrast between the data involving Brazil and Colombia, both South American countries that share a border with each other. Colombia has one of the best indicators related to professional learning. This phenomenon is the result of some measures adopted in the country, such as: the possibility of taking *lato sensu* graduate and master’s degrees since 2015 without the need for a higher education diploma, only with a technical course; the understanding on the part of the Colombian State that the number of professional technicians serves as an instrument to boost the productivity and competitiveness of companies in that country; the entry of qualified personnel into the well-paid job market, which is essential to reduce poverty and improve income distribution; and, finally, actions to increase the creation of strategic partnerships between the productive and educational sectors.

In Colombia, structural changes were adopted in educational policy, linked to employment policy, with a view to reducing youth unemployment levels, with high-quality occupational skills for secondary education, in order to strengthen the competitiveness of that country’s economy^(30)^. Therefore, it is possible to understand that professionalization in the health area is also fundamental to create professional opportunities for adolescents and young people who effectively contribute to the citizenship of this social group, in addition to ensuring the continuous training of professionals for the Brazilian SUS.

### Study limitations

The limitations of this study are related to the possibility of locating other historical sources as well as future discoveries, and the expanded and careful search for documents, which would culminate in future adjustments to this historical narrative.

### Contributions to nursing, health and public policy

This study may contribute to understanding the representation of Nilda Teves’s educational project with CLC. This educational project was a turning point in the history of deinstitutionalization of children in the country, as it managed, in a short period of time, to restructure and give new meaning to the *Centro Piloto de Quintino*, which, for decades, had served to stigmatize children and adolescents in situations of social vulnerability. The project also contributed to the social development of that space and its surroundings, positively impacting the multiple opportunities that full-time education could offer to young people in the region.

In this way, this work also contributed to expanding knowledge of the situation of those who work in this area, in order to promote greater possibility of understanding and appropriation of this approach. This research also serves as an important analytical instrument to broaden the understanding of nursing trajectory in this field.

## FINAL CONSIDERATIONS

The 1990s were marked by a significant reorganization of educational systems to meet the logic of a neoliberal economic policy imposed between the governments of Fernando Collor de Mello and Fernando Henrique Cardoso. This organization prioritized preparing workers according to the job market’ needs, and it was necessary to keep up with global technological changes.

In this context, the country was going through a major economic crisis, with the privatization of large state-owned companies and the cutting of public spending being the government’s main goals at the time. As a result, some federal public administration entities were closed down and dissolved, such as FCBIA, which ceased operations in early 1995. However, society was dissatisfied with the care provided to children and adolescents at social risk at this institution.

To analyze the process of maintaining the nursing assistant course at HCA of the FCBIA Study Center after the extinction of this foundation, in the 1990s, it was necessary to unravel a historical trajectory experienced by two leading actors, Nilda Teves Ferreira and Celso do Nascimento Faustino, whose setting was CLC, at the *Centro Piloto de Quintino*, nortern Rio de Janeiro.

Through the social and political space of CLC, under the coordination of Nilda Teves, and through the efforts of its director, Celso do Nascimento Faustino, professional education in nursing was not interrupted due to the legal contingencies of the closure of FCBIA.

The objective of this study showed that the strategies developed by workers culminated in changes in the *Centro Piloto de Quintino*, with the structuring of CLC and the maintenance of UPAS courses. Thus, it is clear that such strategies resulted in the restructuring and resignification of that space, which was previously intended to stigmatize adolescents in situations of social vulnerability, contributing to making it a space of opportunity for technical professional training in health and nursing.

The study identified, in view of so many setbacks experienced in the social space of the *Centro Piloto de Quintino*, in addition to the legal devices presented, how much education can transform people in the search for opportunities for a more dignified life and how many professionals there were willing to make this difference for so many children and adolescents, opening paths for new studies on this topic.
