# Supplementary material for: Maintaining vocational education: strategies of a public institution aimed at adolescents in social vulnerability
Source: Rev Bras Enferm. 2025 Jun 20;78(2):e20230285. doi: 10.1590/0034-7167-2023-0285 (PMC12185137; doi:10.1590/0034-7167-2023-0285)
Supplement: Translation

# Translation: Manutenção da educação profissionalizante: estratégias de uma instituição pública direcionada ao adolescente em vulnerabilidade social

**DOI:** 10.1590/0034-7167-2023-0285pt

**Authors:** Lilian Dias Ennes, Camila Pureza Guimarães da Silva, Tânia Cristina Franco Santos, Patrícia dos Santos Augusto, Gabriella Picoli dos Santos Faustino, Antonio José de Almeida

**Affiliations:** I Hospital Federal Cardoso Fontes. Rio de Janeiro, Rio de Janeiro. Brasil; II Universidade Federal do Rio de Janeiro. Rio de Janeiro, Rio de Janeiro. Brasil

**Keywords:** Adolescente, Formação Profissional, Educação Profissionalizante, História da Enfermagem, Enfermagem.

## Abstract

**Objetivos:**

analisar as estratégias políticas e administrativas para manutenção dos cursos da Unidade Profissionalizante da Área da Saúde e do Centro de Estudos do Hospital da Criança e do Adolescente da Fundação Centro Brasileiro para Infância e Adolescência.

**Métodos:**

estudo histórico, com fontes constituídas por matérias jornalísticas e documentos oficiais, além de oito participantes. Os dados foram coletados de maio a dezembro de 2021e analisados com base em artigos sobre educação profissionalizante e políticas educacionais de nível nacional e estadual. O recorte temporal compreende os anos de 1990 a 1997.

**Resultados:**

estratégias adotadas por trabalhadores culminaram em mudanças no Centro Piloto de Quintino, com a estruturação do Centro de Educação Integral, e na manutenção dos cursos da Unidade Profissionalizante da Área da Saúde.

**Considerações Finais:**

as estratégias resultaram na reestruturação e ressignificação do espaço que antes se destinava a estigmatizar adolescentes em situação de vulnerabilidade social.

## INTRODUÇÃO

A eleição presidencial de 1989 trouxe um paradoxo para os estudos da política brasileira, ao ser lembrada pela longa jornada de transição democrática do país e pela incerteza no novo cenário eleitoral vivido à época. Fernando Afonso Collor de Mello assumiu a Presidência do Brasil em 15 de março de 1990 (até 1992), sendo esta a primeira eleição presidencial por voto direto, após 21 anos sob a gestão da ditadura militar e quatro anos da promulgação da Constituição da República Federativa do Brasil, ocorrida em 05 de outubro de 1988^(1,2)^. Collor ocupou o cargo de presidente em meio a uma importante crise econômica e social que atravessava o país.

O plano do governo de Fernando Collor para a redução dos índices de inflação incluía metas como ajuste estatal, monetário, fiscal e administrativo, com a proposta de redução da burocracia e do tamanho da máquina pública. A extinção e a dissolução de entidades da administração pública federal, que foram reafirmadas através da Lei nº 8.029, de 12 de abril de 1990, constam como um dos primeiros atos do presidente^(3,4)^.

Assim, o Poder Executivo foi autorizado a extinguir ou transformar algumas entidades públicas vigentes até então. Entre elas, estava a Fundação Nacional do Bem-Estar do Menor (FUNABEM), instituída pela Lei n° 4.513, de 1° de dezembro de 1964, que passou a denominar-se Fundação Centro Brasileiro para Infância e Adolescência (FCBIA), a partir da Lei nº 8.029/1990^(5-7)^.

A FUNABEM foi criada no mesmo ano de implantação do regime militar no Brasil, sendo a política de internação para resolver o problema do menor considerada um marco da instituição, uma vez que tinha como base a doutrina da segurança nacional, empregada pela Escola Superior de Guerra (ESG). Intensos debates foram desencadeados, à época, sobre a formulação de uma entidade de gestão das infâncias e juventudes que viesse a erradicar o antigo Serviço de Assistência ao Menor (SAM), instituição que antecedeu a FUNABEM^(8)^.

A FCBIA, subordinada ao Ministério da Ação Social (MAS), teve como objetivo formular, normatizar e coordenar a política de defesa dos direitos da criança e do adolescente. Esta mudança de designação foi uma estratégia de ressignificação da atenção a esse público, pois havia importante pressão social e dos organismos internacionais, como a influência das diretrizes acerca da criança e do adolescente, promulgadas pela Organização das Nações Unidas (ONU), que impulsionaram essa mudança^(7)^.

Dessa forma, a FCBIA fez parte de um arcabouço de leis que antecederam o Estatuto da Criança e do Adolescente (ECA), delimitado pela Lei nº 8.069, de 13 de julho de 1990^(9)^, que dispõe sobre a proteção integral da criança e adolescente. Antes do ECA, o termo “menor” era empregado aos sujeitos que não possuíam suas necessidades básicas garantidas, e o fator econômico era considerado um dos principais motivos que levaria meninos e meninas ao confinamento em instituições de atendimento, na qual permaneciam por tempo indeterminado^(10)^.

A sede administrativa da FCBIA se encontrava no Rio de Janeiro, por ter sido Distrito Federal por um longo período, mas a fundação possuía diversas unidades, em todo território nacional, de cunho estatal e unidades particulares conveniadas^(7)^. Contudo, após a instauração do ECA, esse modelo socioeducativo de institucionalizar crianças e adolescentes foi enfraquecido.

A percepção das autoridades quanto aos custos de manter essa estrutura precarizada e as constantes críticas da sociedade, que clamava por um atendimento digno aos jovens sob o jugo do Estado, contribuíram para a derrocada da FCBIA, em 1995. Desse modo, Fernando Henrique Cardoso, ao assumir a Presidência da República (1995-1999), extinguiu a FCBIA pela Medida Provisória nº 813, de 1 de janeiro de 1995^(9,10)^.

O encerramento da FCBIA não foi de imediato à tramitação da Medida Provisória nº 813, como publicado pelo jornal Folha de São Paulo, em 02 de janeiro de 1995, em reportagem sob o título: “Legião Brasileira da Boa Vontade e FCBIA deixam de existir de forma gradual”. Era necessário assegurar que os programas e os convênios não fossem interrompidos no processo de transferência de funções a outros ministérios^(10,11)^.

Assim, os profissionais que não estivessem em fase de aposentaria ou não solicitassem a demissão voluntária, à época, seriam transferidos paulatinamente para outros órgãos da esfera federal. As implicações da finalização das atividades da FCBIA repercutiram na vida dos servidores federais, bem como na reorganização de espaços e destinação de patrimônios deixados pela fundação Brasil afora^(10)^.

Cabe destacar que esse contexto teve impacto no Centro de Estudos do Hospital da Criança e do Adolescente (HCA) da FCBIA, setor autorizado a ministrar os cursos profissionalizantes da área da saúde, conforme Parecer nº 52/91 do Conselho Estadual de Educação do Rio de Janeiro (CEE/RJ). Entre os cursos disponibilizados, constavam técnico de enfermagem, auxiliar de enfermagem, auxiliar de patologia clínica, técnico de patologia clínica, atendente de consultório dentário, auxiliarem laboratório de prótese, técnico em laboratório de prótese odontológica e auxiliar de farmácia^(11)^.

No que tange aos cursos profissionalizantes de enfermagem, a despeito do curso técnico de enfermagem estar autorizado, apenas o curso de auxiliar de enfermagem era ministrado, e assim permaneceu, mesmo com o encerramento das atividades da FCBIA. A manutenção do curso de auxiliar de enfermagem se deu após estratégias de enfrentamento adotadas por docentes, discentes e outros trabalhadores da educação, insatisfeitos com o impacto que a interrupção do mesmo traria para os trabalhadores e adolescentes daquele entorno, que percebiam naquela unidade educacional a oportunidade de qualificação profissional. Essa conjuntura resultou na manutenção dos cursos da Unidade Profissionalizante da Área da Saúde (UPAS), efeitos da nova política educacional implementada no Estado do Rio de Janeiro, Brasil, com a criação do Centro de Educação Integral (CEI) no complexo educacional denominado “Centro Piloto de Quintino”.

Diante do exposto, apresentamos a seguinte questão investigativa: como as estratégias adotadas por docentes e autoridades políticas e administrativas impactaram a manutenção dos cursos da UPAS e do Centro de Estudos do HCA da FCBIA após a extinção dessa fundação?

Tal estudo justifica-se pela relevância acadêmica e social que envolve a educação profissionalizante direcionada ao adolescente em vulnerabilidade social, sobretudo quando tal iniciativa é impulsionada, ao mesmo tempo, por políticas educacionais profissionalizantes de âmbito federal e estadual, no caso específico, o estado do Rio de Janeiro.

## OBJETIVOS

Analisar as estratégias políticas e administrativas para a manutenção dos cursos da UPAS e do Centro de Estudos do HCA da FCBIA.

## MÉTODOS

### Aspectos éticos

Os aspectos éticos foram respeitados, conforme as recomendações das Resoluções nº 466/12 e nº 510/16 do Conselho Nacional de Saúde. Para as entrevistas, todos os participantes assinaram um Termo de Consentimento Livre e Esclarecido. O estudo foi aprovado pelo Comitê de Ética em Pesquisa em Pesquisa com Seres Humanos da instituição signatária.

### Tipo de estudo

Trata-se de estudo de abordagem histórica, na perspectiva da história do tempo presente, cujo *corpus* foi composto por fontes históricas diretas (escritas e orais) e indiretas. O recorte temporal compreende os anos de 1990 a 1997, período em que ocorreram publicações de leis relevantes ao contexto deste estudo. É definida como marco histórico inicial a Lei nº 8.029, de 12 de abril de 1990, que alterou a denominação da FUNABEM para FCBIA. A referida lei também modificou sobremaneira a estrutura e o funcionamento do hospital e do Centro de Estudos da FCBIA e, assim, os cursos de qualificação profissional da área da saúde, como o curso de auxiliar de enfermagem. O marco final foi definido em 1997, quando a estrutura física do HCA, parte da extinta FCBIA, incorporou a UPAS. Foram seguidas as diretrizes do *COnsolidated criteria for REporting Qualitative research* (COREQ).

### Cenário do estudo

O cenário da pesquisa foi o Centro Piloto de Quintino, cuja extensão territorial é de 1.900.000 m^2^. Por décadas, este espaço pertenceu ao Governo Federal, sendo cedido, por meio de um convênio, ao Governo do Estado do Rio de Janeiro, que o transformou em um importante polo para desenvolvimento de política pública de educação profissional, sob a gestão do governador Marcello Nunes de Alencar (1995-1999), através da Fundação de Apoio à Escola Pública (FAEP), subordinada à Secretaria Estadual de Educação (SEEDUC), de julho de 1987 a fevereiro de 1996^(12)^.

### Coleta e organização dos dados

A coleta de dados do *corpus* documental ocorreu no período de maio a dezembro de 2021. As fontes históricas diretas escritas foram constituídas por leis, decretos, portarias, projetos de regulamentação do curso de auxiliar de enfermagem, Revista Brasil Jovem e matéria do jornal Folha de São Paulo. A constituição do *corpus* documental se deu através de buscas em páginas virtuais governamentais, nos arquivos institucional, cenário desta pesquisa, e em arquivos pessoais dos participantes deste estudo. As fontes indiretas constituíram-se de artigos sobre a temática.

Os critérios de inclusão das fontes textuais foram documentos que abordassem o cotidiano dos agentes da saúde e da educação que vivenciaram a manutenção do curso de auxiliar de enfermagem do Centro de Estudos do HCA, além daqueles referentes à política educacional no estado do Rio de Janeiro. Os critérios de exclusão foram fontes textuais danificadas por diversos motivos, como umidade, mofo, cupim, traça, entre outros, que tornaram o documento ilegível.

As fontes diretas orais se constituíram de entrevistas com oito participantes, entre os quais estão: pedagogo (1); professor de enfermagem (2); instrutor (1); aluna (1); professor de biologia (1); inspetor de aluno (2). Todos tinham acima de 50 anos, sendo cinco do sexo feminino e três do sexo masculino. Quanto à escolaridade, seis possuíam graduação concluída e, dois, o ensino médio completo. Quanto ao vínculo empregatício, um era concursado federal, cinco, concursados estaduais, um possuía contrato temporário e um não possuía vínculo com a instituição.

A técnica utilizada foi a história oral temática^(13)^. Para a seleção dos participantes, utilizou-se a relação nominal dos profissionais que trabalharam ou estudaram nos cursos profissionalizantes da área da saúde do Centro de Estudos do HCA durante o recorte do estudo. Os critérios de inclusão foram participantes que trabalharam ou estudaram nos cursos profissionalizantes da área da saúde do Centro de Estudos do HCA durante o recorte do estudo. Os critérios de exclusão foram apresentar comprometimento da memória ou outro comprometimento físico ou cognitivo que impedisse a realização da entrevista.

A coleta das entrevistas ocorreu após agendamento via e-mail, ligações telefônicas e busca pelos profissionais em seus respectivos campos de atuação profissional. Apenas após o aceite em participar da pesquisa as entrevistas foram agendadas, de acordo com a disponibilidade de cada participante, com duração entre 40 minutos e 1hora e 30 minutos. Todos os participantes em potencial contatados e que concordaram em participar da pesquisa foram entrevistados.

Cabe destacar que, em função da pandemia do novo coronavírus (SARS-CoV-2 (COVID-19)), e com a intenção de assegurar o distanciamento social para aqueles participantes com dificuldade de transitar ou considerados grupos de risco, as entrevistas foram realizadas e gravadas virtualmente, através de um *link* de acesso ao *Google Meet* enviado aos participantes minutos antes da entrevista. Apenas um participante solicitou que a entrevista acontecesse de forma presencial. Nas duas modalidades de entrevistas, foram lidas e esclarecidas quaisquer dúvidas sobre o Termo de Consentimento Livre e Esclarecido (TCLE), enviado previamente, também de modo virtual, assim como sobre o aceite em participar da gravação proposto pelo estudo.

A fim de garantir o anonimato, os participantes foram identificados pela letra P, seguida de numeral cardinal, conforme a ordem das entrevistas.

### Análise dos dados

Os dados foram organizados, classificados e analisados em conformidade com o método histórico. Para a análise do *corpus* documental, as fontes escritas foram catalogadas em sequência cronológica, e, posteriormente, foi realizada a crítica externa e interna, no sentido de garantir a autenticidade, legitimidade, veracidade e fidedignidade dos documentos. Dessa maneira, a sistemática exposição dos textos historiográficos ao leitor e o uso crítico e analítico dessas se consolidaram como um dos principais fatores constitutivos da afirmação da autenticidade do fato histórico^(14)^.

Quanto às fontes diretas orais, as entrevistas foram transcritas e validadas pelos participantes. A partir de então, o *corpus* documental foi submetido a procedimentos ativos de interrogação dos documentos, com postura independente da versão oficial, possibilitando que se evidenciasse melhor o fenômeno histórico. A confiabilidade dos resultados foi assegurada com a valorização do conjunto documental, e não dos documentos isoladamente, estabelecendo-se os seguintes eixos: As mudanças no Centro Piloto de Quintino com a estruturação do Centro de Educação Integral; A manutenção dos cursos da Unidade Profissionalizante da Área da Saúde no Centro de Educação Integral.

Os dados foram analisados com base em artigos sobre educação profissionalizante e políticas educacionais de âmbito nacional e estadual nas bases de indexação LILACS, MEDLINE, SciELO e BVS Enfermagem, úteis à compreensão do contexto em que as fontes foram produzidas, visando apreender o discurso contido nos textos, ou seja, as fontes indiretas.

## RESULTADOS

### As mudanças no Centro Piloto de Quintino com a estruturação do Centro de Educação Integral

As mudanças políticas e sociais ocorridas no primeiro mandato do presidente Fernando Henrique Cardoso (1995-1999) geraram importantes impactos para o mundo do trabalho, da saúde e da educação, em consonância com o pensamento neoliberal vigente à época, que preconizava a intervenção do Estado na economia, com ampla privatização dos serviços públicos. Ainda que sob críticas de alguns educadores, buscou-se uma intensa reorganização dos sistemas educacionais, que deveriam atender à lógica das demandas do mercado^(4)^.

Entre tais mudanças, esteve o encerramento gradual das atividades da FCBIA na década de 1990. Para isso, porém, o Convênio nº 034-A/00/93, de 10 de agosto de 1993, foi assinado entre o Governo Federal e o Governo do Estado do Rio de Janeiro, com o intuito de assumir um compromisso de cooperação mútua para o desenvolvimento de ações integradas e a concretização de medidas de proteção socioeducativas destinadas às crianças e adolescentes em situação de risco pessoal e social, com foco nas unidades que compõem o complexo educacional denominado “Centro Piloto de Quintino”^(15)^.

Destacou-se, nesse contexto de mudanças, a educadora Nilda Teves Ferreira (1941-2022), autora do projeto e coordenadora do CEI, criado pelo Decreto nº 21.752, de 08 de novembro de 1995, vinculado ao Gabinete Civil. Caberia ao CEI funcionar como a instituição que assumiria as ações socioeducativas da extinta FCBIA, através de proposta de oferta de cursos profissionalizantes integrados a uma prática esportiva, em uma lógica pedagógica bem parecida com a dos Centros Integrados de Educação Pública (CIEP), criados no primeiro mandato do governador Leonel Itagiba de Moura Brizola (1991-1994)^(12,16)^.

Sobre o CEI, Nilda Teves Ferreira confirmou, em entrevista realizada em 2012, e publicada no *International Journal of Phenomenology, HermeneuticsandMetaphysics* em 2022^(17)^, que:

[...] *depois de conquistar os servidores que estavam acostumados ao antigo regime, passamos às obras de adaptações dos prédios e instalações de acordo com o nosso propósito. Pretendíamos edificar ali o que se chamou CEI, Centro de Educação Integral. Tínhamos um grande desafio pela frente, pois todo aquele lugar possuía um imaginário muito negativo... era sombrio, possuía muros e portões altos como uma casa de custódia, tinha árvores com os galhos despencados* [...].

De acordo com a educadora, em artigo de sua autoria, intitulado “Projeto educacional para mega-cidade: relato de caso”, sua experiência na educação teria trazido a firmeza e a autoestima necessárias às mudanças para aquele espaço, denominado pela própria Nilda Teves Ferreira como um “*shopping* pedagógico”, por considerar que ali se vislumbravam inúmeras oportunidades para uma nova geração de crianças e adolescentes, pondo um fim no estigma provocado pelo antigo centro correcional para menores^(17,18)^.

Tal reconhecimento pôde ser observado conforme fragmentos de entrevista dos funcionários do CEI:

[...] *nessa época, tínhamos uma vanguardista da UFRJ, que foi a Nilda Teves. A Nilda Teves foi uma pessoa que acreditou. Ela era uma doutora da Faculdade de Educação da UFRJ e acreditava muito nisso. A Nilda* [Teves] *foi uma das presidentes, a primeira presidente da FAEP que fez todo o arcabouço da FAEP* [...]. (P1)

[...] *eu não tenho palavras para expressar a valorosa contribuição da professora Nilda* [Teves] *para reativação, a revitalização do Centro Piloto de Quintino. Então, minhas palavras para a professora Nilda são de agradecimento* [...]. (P2)

[...] *aquela mulher* [Nilda Teves] *é fantástica* [...] *eu sou fã dela* [...]*fantástica, fantástica, fantástica. O Marcello de Alencar* [governador], *todo mês, ele ia umas quatro ou cinco vezes lá em Quintino só para visitar ela e ver como estavam as coisas por lá. Eles eram muito amigos, muito chegados.* (P3)

[...] *o projeto do CEI da professora Nilda* [Teves] *foi muito bem-vindo, muito bem acolhido pela comunidade, porque eles viram a possibilidade de retomar ações que eles tinham acesso. Como a parte esportiva, por exemplo, e algumas ações pedagógicas.* (P5)

Tinha-se, então, o ambiente político necessário para que Nilda Teves fosse convidada pelo governador Marcello de Alencar para ocupar, entre os anos de 1995 e 1996, o cargo de coordenadora do CEI e, ao mesmo tempo, a presidência da FAEP^(12)^.

Contudo, Nilda Teves Ferreira teve que enfrentar a contrariedade de alguns servidores federais e outros trabalhadores com diferentes vínculos empregatícios, que se sentiram ameaçados. O que motivou a preocupação desses funcionários foi o receio de perder seus vínculos de trabalho, pois eram terceirizados. Havia, ainda, o fato de não aceitarem a saída de crianças e adolescentes que permaneciam internas na transição FCBIA-CEI para outras instituições de acolhimento. Esta situação fez com que os mesmos se organizassem para um enfrentamento diante do que consideravam um descaso das autoridades. Assim, participaram, juntamente com alunos, de um protesto rápido, como foi descrito por uma funcionária contratada:

[...] *o protesto não durou nem meia hora, entendeu? Os alunos sentaram no chão, todo mundo sentou no chão da Rua Clarimundo de Melo, e nós a paralisamos por um período, e a polícia chegou* [...]. *A polícia nos tirou da rua, colocou os alunos para dentro e ainda nos ameaçaram, porque tinha menor e a gente ia responder pelos menores que estavam ali* [...]. (P4)

O vínculo de trabalho precário dos terceirizados - aqueles que não eram funcionários públicos e, portanto, não usufruíam de estabilidade profissional - não os permitiu ir adiante em um movimento de luta pela manutenção do seu posto de trabalho. Sobre esse assunto, Nilda Teves Ferreira confirmou^(17)^ que:

[...] *com o desenvolvimento das escolas e do ensino profissionalizante, os técnicos da instituição chegaram à conclusão* [de] *que não era possível atender cerca de quatro mil alunos e manter os moradores. Tratava-se de uma decisão bastante radical. Ficaria a cargo da Fundação para Infância e Adolescência* [FIA] *a moradia, e o CEI ficaria com a educação, inclusive para os moradores.* [...] *o CEI tornou-se um projeto bem-sucedido, a tal ponto que abriu espaço para agregar-se com as escolas técnicas* [...].

Nesse contexto, a educadora entendia que estava esclarecendo as dúvidas sobre o projeto educacional proposto por ela, que vislumbrava as possibilidades de se criar uma nova instrução de acolhimento pela educação, ao considerar o processo de redemocratização e a necessidade de enfrentamento de toda uma cultura política retrógrada. Nesse sentido, afirmou Nilda Teves Ferreira que:

*Não se passa do velho para o novo de uma vez só! Não se desliza de uma cultura para outra do dia para a noite* ^(17)^.

### A manutenção dos cursos da Unidade Profissionalizante da Área da Saúde no Centro de Educação Integral

Pretendendo instituir maior autonomia administrativa ao CEI, o governador Marcello de Alencar promulgou o Decreto nº 22.011, de 09 de fevereiro de 1996, desvinculando o Centro do Gabinete da Casa Civil e o subordinando à FAEP. Outras sete escolas técnicas estaduais que, até então, eram vinculadas à SEEDUC também foram transferidas para a FAEP. Na ocasião, a FAEP deixou de ser subordinada à SEEDUC para vincular-se à Secretaria Estadual de Ciência e Tecnologia (SECT), o que representou uma importante iniciativa para a educação profissional no Estado^(16,19,20)^. Por esse decreto, alguns profissionais da área da educação que interessavam à gestão do CEI foram nomeados e permaneceram lotados no Centro Piloto de Quintino.

Um dos desafios enfrentados para a gestão do CEI pela educadora Nilda Teves Ferreira foi o fato de ter encontrado, no Centro Piloto de Quintino, cursos da área da saúde, incluindo o de auxiliar de enfermagem, remanescentes do HCA. Alguns desses cursos haviam sido interrompidos em função do encerramento das atividades da FCBIA e da consequente transferência dos seus profissionais para outros órgãos federais. O curso de auxiliar de enfermagem seria retomado em uma área administrativa do CEI, onde estaria a equipe gestora da FAEP/CEI, como descrito por um participante do estudo:

[...] *quando assumimos* [professores concursados], *fomos direcionados então para o Centro de Estudos* [...] *e informados que nós assumiríamos turmas do curso de auxiliares de enfermagem que estavam paralisadas desde que o hospital* [HCA] *havia sido desativado.* [...] *as aulas teóricas aconteceriam em um espaço do prédio na presidência da fundação* [...]. (P5)

Ressalta-se que, de acordo com o Tribunal de Contas da União (TCU 012.096/1996-2)^(21)^, a desativação do HCA já teria sido verificada em 29 de março de 1995. Anteriormente a esse fato, os cursos profissionalizantes da área da saúde eram ministrados nas suas dependências, mais particularmente pelo Centro de Estudos do HCA. Dessa forma, quando os primeiros professores concursados chegassem, eles assumiriam as turmas que estavam nesse prédio administrativo do CEI, já que os professores que os antecediam, enfermeiros lotados no HCA, já haviam sido transferidos ou aposentados.

O conjunto desses cursos ficou conhecido como UPAS. A UPAS se tornou a responsável pela oferta de cursos nos níveis fundamental e médio da área da saúde no Centro Piloto de Quintino. Assim, o pedagogo Celso do Nascimento Faustino (1951-2022), servidor público federal, foi nomeado, conforme o Decreto nº 22.011,1996^(19)^, para assumir a direção da UPAS. Sobre isso, o próprio afirmou:

[...] *houve a modificação na estrutura administrativa da FCBIA* [...] *e os cursos da área da saúde foram transformados e passaram a integrar a Unidade Profissionalizante da Área da Saúde, e esse nome foi dado pela equipe administrativa e pelos professores*[...]. (P2)

O pedagogo Celso do Nascimento Faustino, por ter demonstrado importante capital cultural e científico, além de interesse na manutenção dos cursos profissionalizantes da área da saúde, havia sido convidado pela equipe gestora do CEI, através de Nilda Teves de Ferreira, para assumir a direção da UPAS. Sobre a sua trajetória profissional como servidor público federal junto ao Centro de Estudos do HCA, Celso do Nascimento Faustino relatou:

[...] *pela necessidade de reativar o funcionamento dos cursos de qualificação profissional em nível fundamental e médio na área da saúde do Centro de Estudos* [do HCA], *fui secretário administrativo, passando depois a secretário escolar, coordenador dos cursos e diretor da UPAS em 1996* [...]. (P2)

Comprometido com a UPAS, o pedagogo Celso Faustino aceitou o cargo de diretor, sendo um dos últimos servidores remanescentes do HCA, conhecedor da estrutura e funcionamento dos cursos ofertados. Além disso, a implantação do CEI trouxe maior visibilidade aos cursos oferecidos em todo o Centro Piloto de Quintino, que foram divulgados mais amplamente na gestão de Nilda Teves. Salienta-se que, embora a UPAS fosse reconhecida pelos seus pares desde 1996, apenas em 2001 foi legitimada como unidade de ensino pela Portaria PR/FAETEC nº 79, de 19 de fevereiro^(21-23)^.

Desse modo, entusiasmado com o aumento da procura dos cursos, e por considerar que o curso de auxiliar de enfermagem deveria ser o de maior interesse pela sociedade naquela unidade de ensino, Celso Faustino ponderou com a Nilda Teves a possibilidade de ocupação do hospital, até então desativado, para abrigar a UPAS, de modo definitivo. Assim, o diretor tenta sensibilizar a coordenadora do CEI, conforme fragmento da entrevista:

[...] *eu sempre pressionando a professora Nilda e apelando para ocupar o hospital* [HCA]*, mas eu não vou ocupar tudo, só vou ocupar o primeiro andar e já será possível para organizarmos os cursos* [...]. (P2)

Apesar de ter sido uma reivindicação de professores e alunos, cabe salientar que o diretor da UPAS, a despeito de usufruir de prestígio junto à presidente da FAEP e coordenadora do CEI, responsável por administrar aquela grande área do Centro Piloto de Quintino, entendia que conquistar o espaço do HCA para transformá-lo em unidade de ensino não seria uma tarefa simples. Isso porque ainda permanecia no hospital considerável quantidade de patrimônio federal, o que exigia providências para atender aos entraves burocráticos, com vistas ao remanejamento desses. A despeito do Decreto de 27 de agosto de 1996, registrou-se o encerramento dos trabalhos de inventariança relativa aos bens patrimoniais deixados pela extinta FCBIA^(10,24)^.

Em novembro de 1996, o Decreto nº 2.059 estabeleceu a transferência para o Ministério da Justiça do acervo patrimonial mobiliário da extinta FCBIA, e coube a esse ministério indicar quais seriam os órgãos e entidades contemplados com os mobiliários. Assim, conforme previa o Art. 2º do Decreto nº 2.059, o ministro da Justiça Nelson Azevedo Jobim (1995-1997) autorizou a destinação dos bens móveis que não fossem aproveitados em seus serviços, ou seja, poderiam ser alienados, cedidos ou doados aos estados, ao Distrito Federal e aos municípios^(10,25)^.

O diretor da UPAS teria, ainda, ponderado com Nilda Teves sobre a necessidade da oferta de cursos na área da saúde para os adolescentes da região de Quintino Bocaiúva que possuíssem menor formação, pois conhecia a realidade daquele entorno. Finalmente, Nilda Teves, em 1997, consentiu ao diretor da UPAS ocupar o primeiro andar do prédio do HCA e iniciar a transição dos cursos da área da saúde para aquele espaço.

## DISCUSSÃO

No Brasil, os ideais de uma política neoliberal, iniciada com Fernando Collor de Mello e consolidada com Fernando Henrique Cardoso, deram o tom de uma política que pretendia reduzir gastos públicos através da redução da folha de pagamento dos servidores públicos federais e da privatização de estatais, focos essenciais em seus planos de governo. Para isso, importantes dispositivos legais foram criados para respaldar suas ações, como a Lei nº 8.029, de 12 de abril de 1990, que levou à mudança na denominação de FUNABEM para FCBIA, propondo uma mudança na política de atenção às crianças e adolescentes.

Contudo, o estudo mostrou, na prática, que não foram verificadas transformações significativas na atenção a esse público. Essa mudança na denominação, inicialmente, aplacou um clamor da sociedade em função de tantas críticas que vinham sendo desferidas ao Governo Federal brasileiro pela participação do Brasil na Convenção sobre os Direitos da Criança adotada pela Assembleia Geral da ONU em 20 de novembro de 1989^(26)^.

É possível compreender a ineficácia dessa iniciativa, posto que ainda predominava tanto na sociedade quanto no Estado brasileiro uma percepção distorcida na qual tratava o sujeito que carecia de solidariedade e assistência como alguém que deveria ser alvo de vigilância e repressão. Nesse sentido, as questões de segurança pública relacionadas à menoridade comumente eram associadas a uma parcela da infância e da adolescência que não lograra êxito em razão do seu “abandono escolar, do trabalho informal e/ou precoce ou de uma socialização indevida (como o envolvimento com o consumo e o tráfico de drogas)”. Esse grupo social refletia um sujeito que exigia atenção do Estado, posto que a família não teria cumprido satisfatoriamente sua função de conduzi-lo à vida adulta e à vivência em sociedade, o que justificava, portanto, a intervenção estatal^(8)^.

Essa estrutura social revela e, ao mesmo tempo, consolida um pensamento conservador na sociedade brasileira que contribui para a construção e manutenção de estigmas sociais direcionados a pessoas envolvidas em atos criminosos. Isso, explica em parte, o porquê esse grupo é estigmatizado pela sociedade em geral, que reage e reduz a discussão do problema a temas retrógrados como a diminuição da maioridade penal. Com isso, tem-se que esses jovens são alvo de preconceito e isolamento social^(27)^.

As mudanças políticas e sociais ocorridas no mundo na década de 1990 influenciaram sobremaneira as ações de atenção dos direitos das crianças e adolescentes, advindas de organismos internacionais que ditaram regras pautadas na importância da desinstitucionalização e no cuidado mais digno aos menos favorecidos de uma população infanto-juvenil. No Brasil, em um período marcado pela retomada da ordem constitucional democrática, foi promulgada a Lei n^o^ 8.069 de 1990, conhecida como ECA. O ECA traçava novos paradigmas na condução de um grupo social tão atingido pelas desigualdades estruturais existentes no país^(27)^.

O estudo mostrou a importância do ECA, ao evidenciar dois importantes artigos da Constituição de 1988 para o reconhecimento da criança e do adolescente como sujeitos de direitos: o Art. n^o^227, ao descrever sobre os direitos à vida, à saúde, à alimentação, à educação, ao lazer, à profissionalização, à cultura, à dignidade, ao respeito, à liberdade e à convivência familiar e comunitária; e o Art. n^o^228, que faz importante referência à inimputabilidade penal dos menores de 18 anos, além de ambos serem entendidos como determinantes do dispositivo legal que atendesse às necessidades da criança e do adolescente^(10)^.

Cinco anos mais tarde, Fernando Henrique Cardoso extinguiu a FCBIA através da Medida Provisória nº 813, de 1 de janeiro de 1995. Essa medida provocou, no Rio de Janeiro, local de sua sede, a insatisfação de muitos servidores federais. Como consequência dessa extinção, esses foram, aos poucos, sendo transferidos. O esvaziamento do local desencadeou especulações, pois se tratava de um grande espaço territorial, de aproximadamente 1.900.000 m^2^, conhecido como Centro Piloto de Quintino. Ademais, outros fatores que levaram ao encerramento da FCBIA e das instituições conveniadas a ela por todo o país foram o alto custo de manutenção, as suspeitas de fraude e corrupção, além da desaprovação social que a instituição vinha causando^(10)^.

A despeito de como se configurou o Centro Piloto de Quintino, cabe destacar que se transformou em um importante polo de educação profissional pela articulada e competente educadora Nilda Teves Ferreira, que enfrentou o descrédito de alguns, mas conseguiu, por meio do apoio político do governador Marcello Alencar, desenvolver o projeto intitulado de CEI, criado pelo Decreto nº 21.752, de 08 de novembro de 1995, vinculado, inicialmente, ao Gabinete Civil.

Nilda Teves Ferreira pretendia transformar aquele cenário, que mantinha, no imaginário coletivo de profissionais, alunos e população do entorno, lembranças estigmatizantes de agruras vivenciadas por crianças e adolescentes que outrora estiveram naquele espaço. Para tanto, é importante considerar o reconhecimento dos diferentes agentes que atuaram com Nilda Teves, percebendo-a como uma referência no campo da educação no estado do Rio de Janeiro.

O pedagogo Celso do Nascimento Faustino, servidor federal que conhecia todo o histórico do Centro de Estudos da FCBIA, por ter atuado como secretário administrativo na fase em que o HCA e o Centro de Estudos - estruturas vinculadas à FCBIA - ainda estavam ativos, foi convidado e nomeado como diretor da UPAS pela diretora do CEI à época. Esse servidor também se destacou por ter protagonizado a defesa pela continuidade de cursos profissionalizantes da área da saúde, incluindo o de auxiliar de enfermagem, considerado por ele o mais relevante dos cursos da UPAS.

Cabe salientar que, no período de mudança de denominação de FUNABEM para FCBIA até o encerramento de suas atividades em 1995, os cursos que eram realizados nas dependências do HCA deixaram de ser realizados. Nesse cenário, o pedagogo Celso do Nascimento Faustino, além de professores e alunos da UPAS, envidaram esforços para a continuidade dos cursos e suas realizações nas dependências do HCA, que já se encontravam desativadas. Nesse espaço, permaneciam todos os bens patrimoniais e documentação pertinente ao período em que esteve ativo. Essa guarda dificultava a sua ocupação.

O entendimento da importância da ocupação do antigo prédio - que por muito tempo abrigou o HCA - ensejou uma luta dos servidores para que, nesse espaço, pudessem funcionar os cursos da área da saúde. O local era considerado o mais apropriado para a formação profissional de níveis fundamental e médio na área da saúde, e sua aquisição representava um bem simbólico para os profissionais dos diferentes cursos.

Mesmo contando com prestígio de Nilda Teves, o pedagogo Celso Faustino sabia que liberar o espaço do HCA não seria fácil, em virtude de todos os bens patrimoniais que haviam sido deixados em seu interior. Embora um decreto de 27 de agosto de 1996 registrasse o encerramento dos trabalhos de inventariança relativa a esses bens, o novo Decreto nº 2.059, de 05 de novembro de 1996, designou a transferência para o Ministério da Justiça do acervo patrimonial mobiliário da extinta FCBIA, cabendo-lhe indicar quais seriam os órgãos e entidades contemplados com os mobiliários^(10,25)^.Finalmente, em 1997, Nilda Teves autorizou o diretor da UPAS a ocupar o primeiro andar do prédio do HCA e a iniciar a transição dos cursos da área da saúde para aquele espaço.

Com isso, tinha-se a retomada das atividades educacionais voltadas para a profissionalização de adolescentes e jovens no Centro Piloto de Quintino e, por conseguinte, o resgate da perspectiva de profissionalização daquele grupo. Para melhor compreender a relevância dessa iniciativa, cabe resgatar a assinatura da Lei nº 8.080, de 19 de setembro de 1990, que implementou a Sistema Único de Saúde (SUS) no Brasil, o que assegurava a atenção integral à saúde enquanto um direito de todos os brasileiros. Sobre o curso de auxiliar de enfermagem, um dos mais procurados pelos jovens estudantes do Centro Piloto de Quintino, a busca por qualificação profissional junto ao mercado de trabalho era justificada também em função da contribuição na manutenção e desenvolvimento do SUS, pois se trata do maior contingente entre os profissionais de saúde em atuação no Brasil^(28,29)^.

A importância da qualificação profissional para adolescentes e jovens é exitosa em outros países que adotaram iniciativas de profissionalização voltadas para esse grupo populacional enquanto política de desenvolvimento social. Estudos envolvendo 40 países e coordenados pela Organização para a Cooperação e Desenvolvimento Econômico (OCDE), em 2013, evidenciam que o Brasil se situava entre os países com baixa participação de jovens em cursos de educação profissionalizante. Dados de 2012 revelam que,entre os estudantes inseridos no sistema educacional de nível médio (geral ou técnico), 14% eram matriculados na Educação Profissional e Tecnológica (EPT) e, em 2015, apenas 9%. Entretanto, em outros países membros da OCDE, essa porcentagem chega a 70% de jovens inseridos no programa de EPT^(30)^.

Ainda de acordo com esse estudo da OCDE, é possível evidenciar um contraste importante entre os dados envolvendo o Brasil e a Colômbia, ambos os países da América do Sul e que fazem fronteira entre si. A Colômbia ostenta um dos melhores indicadores relacionados à aprendizagem profissional. Esse fenômeno é resultado de algumas medidas adotadas no país como: a possibilidade de cursar, desde 2015, pós-graduação *lato sensu* e mestrado sem a necessidade de um diploma de curso superior, apenas com o curso técnico; o entendimento por parte do Estado colombiano de que a quantidade de técnicos profissionais funciona como instrumento para impulsionar a produtividade e a competitividade das empresas daquele país; o ingresso de pessoal capacitado no mercado de trabalho bem remunerado fundamental para diminuir a pobreza e melhorar da distribuição de renda; e, por fim, ações para incrementar a criação de parcerias estratégicas entre o setor produtivo e o educacional.

Na Colômbia, foram adotadas mudanças estruturais na política educacional articuladas com a política de emprego, com vistas a reduzir os níveis de desemprego juvenil, com habilidades ocupacionais de alta qualidade para o nível médio, a fim de reforçar a competitividade da economia daquele país^(30)^. Logo, é possível compreender que a profissionalização na área da saúde é também fundamental para criar oportunidade profissional para adolescentes e jovens que contribuem efetivamente para a cidadania desse grupo social, além de assegurar a formação contínua de profissionais para o SUS brasileiro.

### Limitações do estudo

As limitações do estudo estão relacionadas à possibilidade de localização de outras fontes históricas, assim como descobertas futuras, e à busca de maneira ampliada e criteriosa dos documentos, o que culminaria em ajustes futuros nessa narrativa histórica.

### Contribuições para as áreas da enfermagem, saúde e políticas públicas

Este estudo poderá colaborar para o entendimento acerca da representação do projeto educacional de Nilda Teves com o CEI. Esse projeto educacional foi um divisor de águas no que tange à história da desinstitucionalização de crianças no país, por conseguir, em pouco tempo, reestruturar e ressignificar o Centro Piloto de Quintino, que, por décadas, teria servido para estigmatizar crianças e adolescentes em situação de vulnerabilidade social. O projeto também contribuiu para o desenvolvimento social daquele espaço e do seu entorno, impactando positivamente as múltiplas oportunidades que o ensino integral poderia oferecer aos jovens da região.

Dessa forma, este trabalho também contribuiu para ampliação do conhecimento da conjuntura dos que atuam nessa área, a fim de promover maior possibilidade de entendimento e apropriação dessa abordagem. Esta pesquisa também serve de importante instrumento analítico para ampliar a compreensão da trajetória da enfermagem nesse campo.

## CONSIDERAÇÕES FINAIS

A década de 1990 foi marcada por uma expressiva reorganização dos sistemas educacionais para atender à lógica de uma política econômica neoliberal imposta entre os governos de Fernando Collor de Mello e de Fernando Henrique Cardoso. Tal organização priorizava preparar os trabalhadores conforme a necessidade do mercado de trabalho, e era preciso acompanhar as mudanças tecnológicas mundiais.

Nesse contexto, o país atravessava uma importante crise econômica, sendo a privatização de grandes estatais e o corte de gastos públicos as principais metas do governo da época. Assim, ocorreram a extinção e a dissolução de algumas entidades da administração pública federal, como foi o caso da FCBIA, que teve as suas atividades encerradas no início de 1995. Não obstante, a sociedade estava insatisfeita com o atendimento às crianças e adolescentes em risco social nessa instituição.

Para analisar o processo de manutenção do curso de auxiliar de enfermagem do HCA do Centro de Estudos da FCBIA após a extinção dessa fundação, na década de 1990, foi necessário o deslindar de uma trajetória histórica vivenciada por dois protagonistas, Nilda Teves Ferreira e Celso do Nascimento Faustino, cujo cenário foi o CEI, no Centro Piloto de Quintino, na zona norte do Rio de Janeiro.

Através do espaço social e político do CEI, sob coordenação de Nilda Teves, e pelo empenho de seu diretor, Celso do Nascimento Faustino, a educação profissional em enfermagem não foi interrompida devido às contingências legais do encerramento da FCBIA.

O objetivo do estudo evidenciou que as estratégias desenvolvidas por trabalhadores culminaram em mudanças no Centro Piloto de Quintino, com a estruturação do CEI e a manutenção dos cursos da UPAS. Assim, tem-se que tais estratégias resultaram na reestruturação e ressignificação daquele espaço, que antes se destinava a estigmatizar adolescentes em situação de vulnerabilidade social, contribuindo para torná-lo um espaço de oportunidade de formação profissional técnica na saúde e na enfermagem.

O estudo identificou, diante de tantas revezes experienciadas no espaço social do Centro Piloto de Quintino, além dos dispositivos legais apresentados, o quanto a educação pode transformar pessoas na busca de oportunidades por uma vida mais digna e o quanto existiram profissionais dispostos a fazer essa diferença para tantas crianças e adolescentes, abrindo caminhos para novos estudos sobre esta temática.
